# Comparative Effectiveness of Cladribine and S1P Receptor Modulators in Treatment-Naive Relapsing-Remitting MS

**DOI:** 10.1001/jamanetworkopen.2025.41025

**Published:** 2025-11-03

**Authors:** Shalom Haggiag, Luca Prosperini, Massimo Filippi, Maria A. Rocca, Pietro Iaffaldano, Francesco Patti, Matilde Inglese, Giovanna Borriello, Rocco Totaro, Giacomo Lus, Roberta Fantozzi, Vincenzo Brescia Morra, Silvia Romano, Jessica Frau, Girolama Alessandra Marfia, Giorgia Teresa Maniscalco, Maria Pia Amato, Alessia Di Sapio, Giovanna De Luca, Sebastiano Giuseppe Crisafulli, Erica Curti, Matteo Foschi, Paola Cavalla, Giuseppe Salemi, Antonella Conte, Paola Valentino, Diana Ferraro, Alessandra Lugaresi, Sabrina Realmuto, Paola Perini, Elisabetta Ferraro, Sara Montepietra, Carlo Avolio, Marika Vianello, Paola Gazzola, Fabiana Marinelli, Livia Pasquali, Sebastiano Bucello, Domizia Vecchio, Alessandra Protti, Francesca Sangalli, Marco Rovaris, Luigi Grimaldi, Milena De Riz, Paolo Barone, Valentina Scarano, Bonaventura Ardito, Leonardo Sinisi, Paolo Immovilli, Ilaria Pesci, Elena Colombo, Marco Alfonso Capobianco, Cristina Fioretti, Maria Gabriella Coniglio, Antonello Giordano, Tiziana Tassinari, Daniela Cargnelutti, Francesca Matta, Mario Falcini, Maurizia Gatto, Nerina Mascoli, Roberto Balgera, Edoardo Sessa, Rosa Iodice, Claudio Solaro, Katrin Plewnia, Mario Santangelo, Valeria Barcella, Maria Teresa Ferrò, Francesco Sica, Raffaella Cerqua, Giuseppe Santuccio, Francesco Corea, Alessandro Leone, Davide Nasuelli, Augusto Maria Rini, Giampaolo Brichetto, Salvatore Cottone, Monica Ulivelli, Matteo Pizzorno, Patrizia Rossi, Eva Milano, Luigi Zuliani, Serena Ruggieri, Claudio Gasperini, Maria Trojano, Carla Tortorella

**Affiliations:** 1 Centro Sclerosi Multipla, Dipartimento di Neuroscienze, Azienda Ospedaliera San Camillo-Forlanini, Rome, Italy; 2 Neurology Unit, Istituto di Ricovero e Cura a Carattere Scientifico (IRCCS) San Raffaele Scientific Institute, Vita-Salute San Raffaele University, Milan, Italy; 3 Centro Sclerosi Multipla, DiBraiN: Dipartimento di Biomedicina Traslazionale e Neuroscienze, Università di Bari, Bari, Italy; 4 Dipartimento G.F. Ingrassia, Scienze Mediche e Chirurgiche e Tecnologie Avanzate, Università Catania; Unita Operativa Semplice Sclerosi Multipla, Azienda Ospedaliero Universitaria Policlinico G. Rodolico, San Marco, Università Catania Italy; 5 Department of Neuroscience, Rehabilitation, Ophthalmology, Genetics, Maternal and Child Health (DINOGMI), University of Genoa, Istituto di Ricovero e Cura a Carattere Scientifico (IRCCS) Ospedale Policlinico San Martino, Genoa, Italy; 6 Centro Clinico Sclerosi Multipla, Ospedale Fatebenefratelli San Pietro, Rome, Italy; 7 Centro Malattie Demielinizzanti, Clinica Neurologica Ospedale San Salvatore, L’Aquila, Italy; 8 Centro Clinico per la Sclerosi Multipla, II Clinica Neurologica, II Università di Napoli, Naples, Italy; 9 Centro Sclerosi Multipla - Istituto di Ricovero e Cura a Carattere Scientifico (IRCCS) Neuromed, Pozzilli, Italy; 10 Centro Regionale Sclerosi Multipla, Unità Operativa Semplice, Azienda Ospedaliero Universitaria, Policlinico Federico II, Naples, Italy; 11 Department of Neurosciences, Mental Health and Sensory Organs, Sapienza University of Rome, Rome, Italy; 12 Multiple Sclerosis Center, Azienda Sanitaria Locale di Cagliari Department of Medical Science and Public Health, University of Cagliari, Cagliari, Italy; 13 Unita Operativa Semplice Dipartimentale Sclerosi Multipla, Dipartimento Medicina dei Sistemi, Università Tor Vergata, Rome, Italy; 14 Azienda Ospedaliera di Rilievo Nazionale Antonio Cardarelli, Naples, Italy; 15 Dipartimento NEUROFARBA, Sezione Neuroscienze, Università degli Studi di Firenze, Centro Sclerosi Multipla SODc Riabilitazione Neurologica, Azienda Ospedaliero Universitaria Careggi, Florence, Italy; 16 Regional Referral Multiple Sclerosis Centre, Department of Neurology, University Hospital San Luigi Gonzaga, Orbassano, Italy; 17 Centro Sclerosi Multipla, Clinica Neurologica Policlinico SS. Annunziata, Chieti, Italy; 18 Divisione di Neuroimmunologia e Malattie Neuromuscolari, Centro Sclerosi Multipla, Istituto Neurologico Carlo Besta, Milan, Italy; 19 Centro Sclerosi Multipla, Unita Operativa Complessa di Neurologia, Dipartimento di Medicina Generale e Specialistica, Azienda Ospedaliero Universitaria di Parma, Parma, Italy; 20 Department of Biotechnological and Applied Clinical Sciences, University of L’Aquila, L’Aquila, Italy; 21 Department of Neuroscience, Multiple Sclerosis Center, Neurology Unit, S. Maria della Croci Hospital AUSL Romagna, Ravenna, Italy; 22 Centro Sclerosi Multipla, Dipartimento di Neuroscienze, Università di Torino e Azienda Ospedaliero Universitaria Città della Salute e della Scienza di Torino, Turin, Italy; 23 Dipartimento di Radiologia, Diagnostica, Interventistica e Stroke, Azienda Ospedaliera Universitaria Policlinico Paolo Giaccone, Palermo, Italy; 24 Department of Human Neurosciences, Sapienza University of Rome, Rome, Italy; 25 Isituto di Ricovero e Cura a Carattere Scientifico Neuromed, Pozzilli (IS), Italy; 26 Centro Sclerosi Multipla, Policlinico Universitario Magna Graecia, Catanzaro, Italy; 27 Dipartimento di Neuroscienze, Ospedale Civile di Baggiovara, Azienda Ospedaliero-Universitaria, Modena, Italy; 28 Dipartimento di Scienze Biomediche e Neuromotorie, Università di Bologna, Bologna, Italy; 29 Isituto di Ricovero e Cura a Carattere Scientifico, Istituto delle Scienze Neurologiche di Bologna, Bologna, Italy; 30 Centro Sclerosi Multipla, UOC di Neurologia con Stroke Unit, Azienda Ospedaliera Ospedali Riuniti Villa Sofia-Cervello, Palermo, Italy; 31 Centro Sclerosi Multipla, UOC clinica neurologica, Azienda Ospedale Università di Padova; 32 Centro Sclerosi Multipla, Ospedale San Filippo Neri, ASL Roma 1, Rome, Italy; 33 Centro Sclerosi Multipla, UOC Neurologia, Arcispedale Santa Maria Nuova, AUSL Reggio Emilia, Reggio Emilia, Italy; 34 Centro Interdipartimentale per le Malattie Demielinizzanti, SC Neurologia Universitaria, Azienda Ospedaliero Universitaria Policlinico Foggia, Foggia, Italy; 35 Centro Sclerosi Multipla, UOC Neurologia, Ospedale Ca’ Foncello, ULSS2 Marca Trevigiana, Treviso, Italy; 36 SC Neurologia - Centro Sclerosi Multipla, Ospedale Padre Antero Micone, ASL3 Genovese, Genoa, Italy; 37 UOC Neurologia, Centro Sclerosi Multipla, Ospedale Fabrizio Spaziani, Frosinone, Italy; 38 Centro Malattie Demielinizzanti UO Neurologia, Dipartimento di Medicina Clinica e Sperimentale, Università di Pisa, D.A.I. Neuroscienze AOUP, Pisa, Italy; 39 UOSD Neurologia, Ospedale E. Muscatello Augusta ASP8, Augusta (SR), Italy; 40 Centro Sclerosi Multipla, Clinica Neurologica, Dipartimento di Medicina Traslazionale, Università del Piemonte Orientale, Novara, Italy; 41 ASST Grande Ospedale Metropolitano Niguarda, Milan, Italy; 42 Centro ad Alta Specializzazione per la diagnosi e la cura della Sclerosi Multipla, Ospedale Valduce, Como, Italy; 43 Fondazione Don Carlo Gnocchi Isituto di Ricovero e Cura a Carattere Scientifico, Milan, Italy; 44 UOC Neurologia and Centro Sclerosi Multipla, Fondazione Istituto G. Giglio, Cefalù, Italy; 45 UniCamillus–Saint Camillus International University of Health Sciences, Rome, Italy; 46 Fondazione Isituto di Ricovero e Cura a Carattere Scientifico Ca’ Granda Ospedale Maggiore Policlinico, Milan, Italy; 47 Azienda Ospedaliera San Giovanni di Dio e Ruggi, Salerno, Italy; 48 AORN San Giuseppe Moscati, Avellino, Italy; 49 Ospedale Della Murgia Fabio Perinei, Bari, Italy; 50 UOC Neurologia, Centro Sclerosi Multipla, Ospedale San Paolo, ASL Napoli 1 Centro, Naples, Italy; 51 Azienda Unità Sanitaria Locale di Piacenza, Ospedale Guglielmo da Saliceto, Piacenza, Italy; 52 Ospedale di Vaio, AUSL Parma, Fidenza, Italy; 53 Multiple Sclerosis Centre, Isituto di Ricovero e Cura a Carattere Scientifico Mondino Foundation, Pavia, Italy; 54 Azienda Ospedaliera S. Croce e Carle, Cuneo, Italy; 55 Spedali Riuniti, Livorno, Italy; 56 Ospedale Madonna delle Grazie, Matera, Italy; 57 ASP Ragusa, Ospedale R. Guzzardi, Ragusa, Italy; 58 Ospedale Santa Corona, Pietra Ligure, Italy; 59 Azienda Sanitaria Universitaria Friuli Centrale Ospedale Santa Maria della Misericordia, Udine, Italy; 60 Ospedale Garibaldi Centro, Catania, Italy; 61 Ospedale di Prato, Prato, Italy; 62 Ospedale Generale Regionale F. Miulli, Acquaviva delle Fonti, Italy; 63 Azienda Socio Sanitaria Territariale Lariana Ospedale S. Anna, Como, Italy; 64 Azienda Ospedaliera A. Manzoni, Lecco, Italy; 65 Centro Neurolesi Bonino Pulejo Isituto di Ricovero e Cura a Carattere Scientifico, Messina, Italy; 66 Università di Napoli Federico II, Naples, Italy; 67 Struttura Complessa di Neurologia, Ospedale Galliera, Genoa, Italy; 68 Azienda USL Toscana Sud Est, Ospedale Misericordia, Grosseto, Italy; 69 Ospedale Ramazzini, Carpi, Italy; 70 Azienda Socio Sanitaria Territariale Papa Giovanni XXIII, Bergamo, Italy; 71 Neuroimmunology, Center for Multiple Sclerosis, Azienda Socio Sanitaria Territariale Crema, Crema, Italy; 72 ASL Latina Ospedale Sclerosi Multipla Goretti via Lucia Scaravelli, Latina, Italy; 73 Clinica Neurologica, Azienda Ospedaliero Universitaria delle Marche, Ancona, Italy; 74 Ospedale San Carlo, Azienda Socio Sanitaria Territariale Santi Paolo e Carlo, Milan, Italy; 75 Ospedale San Giovanni Battista, Foligno, Italy; 76 Azienda Socio Sanitaria Territoriale della Valtellina e Alto Lario, Sondrio, Italy; 77 Azienda Socio Sanitaria Territariale della Valle Olona, Ospedale di Saronno, Saronno, Italy; 78 Ospedale A. Perrino, Brindisi, Italy; 79 Servizio di Riabilitazione AISM Liguria, Genoa, Italy; 80 Azienda di Rilievo Nazionale ed Alta Specializzazione Ospedali Civico Di Cristina Benfratelli, Palermo, Italy; 81 Università degli Studi di Siena, Siena, Italy; 82 Ospedale San Paolo, Savona, Italy; 83 Ambulatorio Sclerosi Multipla e Malattie Demielinizzanti del SNC, UOC di Neurologia, Ospedale San Bassiano AULSS7, Bassano del Grappa, Italy; 84 Ospedale Maria Vittoria, Turin, Italy; 85 Ospedale San Bortolo, Vicenza, Italy

## Abstract

**Importance:**

Early treatment choice in relapsing-remitting multiple sclerosis (RRMS) is prognostically crucial, yet robust comparative data on cladribine vs sphingosine-1-phosphate receptor modulators (S1PRMs) in treatment-naive patients with RRMS are limited.

**Objective:**

To compare the clinical effectiveness of cladribine vs S1PRMs in treatment-naive individuals with RRMS.

**Design, Setting, and Participants:**

This comparative effectiveness research study used data from 108 Italian multiple sclerosis (MS) centers affiliated with the Italian Multiple Sclerosis and Related Disorders Register. All treatment-naive patients with RRMS who initiated cladribine or an S1PRM (fingolimod, ozanimod, or ponesimod) between January 2011 and October 2021 and had at least 12 months of follow-up were included. Propensity score matching and pairwise censoring were used to balance baseline differences and follow-up duration. Patient data were extracted from the register in September 2024.

**Exposure:**

Initiation of cladribine or an S1PRM, with duration reflecting clinical practice.

**Main Outcomes and Measures:**

The primary outcome was no evidence of disease activity (NEDA-3) and its subcomponents. Secondary analyses evaluated disability accrual subdivided into progression independent of relapse activity (PIRA) and relapse-associated worsening (RAW), plus variables associated with treatment response. Cox proportional hazards models, adjusted for visit and magnetic resonance imaging (MRI) frequency, were used to compare outcomes.

**Results:**

Of the 1587 patients (485 taking cladribine and 1102 taking S1PRMs), matching yielded 475 pairs (950 individuals; mean [SD] age, 34.7 [10.7] years; 686 female [72.2%]), with a median (IQR) follow-up period of 25 (12-60) months. For the cladribine vs S1PRM groups, no significant differences were observed in relapse rates (72 patients [15.2%] vs 76 patients [16.0%]), MRI activity (137 patients [31.3%] vs 145 patients [34.8%]), or NEDA-3 loss (194 patients [44.4% vs 219 patients [52.2%]). Cladribine was associated with a lower risk of disability worsening vs S1PRM (54 patients [11.4%] vs 70 patients [14.7%]; hazard ratio [HR], 0.64; 95% CI, 0.42-0.96; *P* = .03), a finding that was confirmed in sensitivity analyses for patients younger than 40 years, those whose diagnoses were made according to the 2017 McDonald Criteria, and those with Expanded Disability Status Scale score less than or equal to 3.0. This was mainly driven by reduced PIRA risk with cladribine (HR, 0.40; 95% CI, 0.20-0.79; *P* = .009), with no RAW difference. After 36 months, patients treated with cladribine showed higher relapse risk (HR, 1.81; 95% CI, 1.02-3.20; *P* = .04) and increased NEDA-3 loss (HR, 2.08; 95% CI, 1.18-3.67; *P* = .01). Discontinuation rates were similar (HR, 0.92; 95% CI, 0.67-1.15; *P* = .58).

**Conclusions and Relevance:**

These findings suggest cladribine was associated with superior effectiveness in reducing disability progression over 25 months, likely due to reduced PIRA, despite comparable short-term NEDA-3 outcomes. However, relapse prevention declined after 36 months, suggesting retreatment or therapy modification within 3 years may be needed to maintain long-term disease control.

## Key Points

**Question:**

How does cladribine compare with sphingosine-1-phosphate receptor modulators (S1PRMs) in treatment-naive patients with relapsing-remitting multiple sclerosis?

**Findings:**

In this comparative effectiveness research study of 950 propensity score–matched treatment-naive patients with at least 12 months (median, 25 months) of follow-up, cladribine and S1PRMs demonstrated comparable relapse rates, magnetic resonance imaging activity, and no evidence of disease activity with 3 components loss, with cladribine associated with a significantly lower risk of confirmed disability worsening, likely driven by a reduction in progression independent of relapse activity events, and with indications of reduced clinical activity control beyond 36 months.

**Meaning:**

These findings suggest cladribine may provide greater short-term protection against disability progression, with a possible need for redosing or treatment switch to sustain disease control beyond 3 years.

## Teaser

This comparative effectiveness research study investigates the clinical effectiveness of cladribine vs sphingosine-1-phosphate (S1P) receptor modulators in treatment-naive patients with relapsing-remitting multiple sclerosis (RRMS).

## Introduction

Multiple sclerosis (MS) is a chronic autoimmune disease with inflammatory and neurodegenerative components, requiring long-term therapy to control inflammation and delay progression. These processes may start before the first clinical event, highlighting the importance of early treatment.^1^

Early initiation of high-efficacy, disease-modifying therapies (DMTs) reduces disability, improves quality of life, and lowers socioeconomic burden.^2,3,4,5,6,7,8^ Although treatment has traditionally followed an escalation approach, evidence supports earlier use of high-efficacy agents.^6,9,10,11^ Choosing the optimal DMT at diagnosis remains challenging due to prognostic uncertainty, differences in efficacy and safety, lack of head-to-head data, and constraints from guidelines and reimbursement policies.^12,13^

Among oral DMTs for relapsing-remitting MS (RRMS), sphingosine-1-phosphate receptor modulators (S1PRMs)^14,15,16^ and cladribine tablets^17,18^ show higher efficacy than placebo and lower-efficacy agents, but in many health care systems, including the European Union, their use is largely limited to patients for whom prior therapies failed or those with highly active disease.^13^ Empirical evidence suggests that early use of cladribine or S1PRMs, either first-line or early in the disease, may provide greater benefit than delayed initiation,^19,20,21,22,23,24,25,26,27^ with lower switching rates in treatment-naive patients with RRMS.^28^ Some observational studies indicate cladribine may yield better persistence and lower relapse rates than fingolimod, while others report comparable outcomes.^29,30,31,32,33^ One analysis found cladribine superior for disability improvement.^33^ Comparative data with ozanimod and ponesimod, especially in treatment-naive patients with RRMS, are lacking.

To address this gap, we assessed the effectiveness of cladribine vs S1PRMs specifically in patients with treatment-naive RRMS, using the Italian Multiple Sclerosis and Related Disorders Register.^34^ A propensity score (PS)–matched design compared relapse risk, magnetic resonance imaging (MRI) activity, and disability progression, aiming to inform early therapeutic strategies.

## Methods

### Patients

This comparative effectiveness research study included individuals aged 18 to 65 years with RRMS who started cladribine or a S1PRM—fingolimod, ozanimod, or ponesimod—as their first DMT. Patients were required to have at least 1 year of follow-up. Those who received a diagnosis before the 2001 McDonald Criteria^35^ or with secondary progressive MS were excluded; accordingly, individuals treated with siponimod were not considered, as it is currently approved only for active secondary progressive MS in Italy. Demographic and clinical information, including disease history, was collected. MRI data, specifically, the presence of gadolinium-enhancing lesions in the year prior to treatment initiation and during the follow-up, as well as the development of new lesions on T2-weighted MRI during follow-up, were also obtained when available. Data on treatment discontinuation and subsequent therapies were also retrieved.

Patient data were extracted in September 2024 from the Italian Multiple Sclerosis and Related Disorders Register.^48^ This study was approved by the ethics committee of the Azienda Ospedaliero-Universitaria Policlinico of Bari as well as by the local ethics committees in all participating centers. All patients provided written informed consent for inclusion and use of anonymized data. Reporting followed International Society for Pharmacoeconomics and Outcomes Research (ISPOR) reporting guidelines.

### Study Outcomes

As study outcomes, we considered the proportions of patients who experienced relapses, disability worsening, MRI activity, or any of the aforementioned types of disease activity. Accordingly, we also calculated their counterparts—that is, the proportions of patients who reached the NEDA-3 status, a combined measure defined as the absence of clinical relapses, disability worsening, and MRI activity.

A relapse was defined as any new neurological symptom not associated with fever or infection lasting more than 24 hours and accompanied by new neurological signs. Disability worsening was defined as a 1.5-point increase (if baseline Expanded Disability Status Scale [EDSS] score was 0), 1.0-point increase (if baseline EDSS score was <5.5), or 0.5-point increase (if baseline EDSS score was ≥5.5) confirmed 6 months apart; patients with worsening near the end of follow-up received additional assessment to confirm the outcome. MRI activity was defined as new gadolinium-enhancing lesions on contrast-enhanced, T1-weighted images or new and/or enlarged lesions on T2-weighted images relative to baseline and/or previous scan.

### Statistical Analysis

Baseline characteristics collected included sex, age, calendar year, location of onset (optic nerve vs others^36^), time since first symptom, EDSS score, number of relapses, and annualized relapse rate (ARR) up to treatment initiation. Baseline MRI data were excluded due to the high rate of missing data, particularly among patients taking S1PRMs (eTable 1 in Supplement 1). Differences in baseline characteristics between the cladribine and S1PRMs groups were assessed using Fisher exact test or the Mann-Whitney *U* test, as appropriate.

Since treatment allocation was not randomized, we performed 1:1 matching using a combination of both PS-based nearest-neighbor matching within a 0.1 caliper (without replacement) and exact matching on sex and location of onset. PS values were estimated by logistic regression, including the above covariates, with treatment group as the dependent variable. Balance was evaluated with standardized differences (Cohen *d* > 0.20 denotes imbalance).

To account for differences in follow-up, we applied pairwise censoring, right-censoring each pair at the shorter follow-up time. Combined with PS matching, this yielded comparable baseline features and follow-up duration. Analyses were conducted on matched samples with Cox proportional hazards models, stratified by pairs and adjusted for visit frequency. The observation period extended from baseline to last visit or outcome. To address therapeutic lag, relapses and disability worsening within the first 3 and 6 months, respectively, were excluded.

To test robustness, we performed sensitivity analyses in subgroups: (1) age younger than 40 years at treatment initiation; (2) diagnosis per 2017 revised McDonald Criteria^37^; (3) baseline EDSS score less than or equal to 3.0; (4) treatment with fingolimod (the most prescribed S1PRM), compared with matched counterparts; (5) presence or absence of baseline gadolinium-enhancing lesions (requiring new PS-matching and censoring); and (6) posttreatment follow-up duration greater than or equal to 36 months. The first 5 subgroups reflect demographic and clinical characteristics that are commonly encountered in current clinical practice and/or are associated with distinct prognostic implications.^38,39^ The final subgroup was selected to assess the durability of cladribine’s effectiveness beyond the treatment administration period.

We also explored associations between baseline variables (excluding treatment) and risk of disease activity using Cox models within each treatment group, adjusting for visit and MRI frequency. To investigate whether differences in worsening of disability between treatments were primarily driven by progression independent of relapse activity (PIRA) or relapse-associated worsening (RAW), we conducted separate Cox regression analyses on these subgroups. PIRA events were defined as confirmed disability progression in patients who did not experience any clinical relapses during the entire follow-up period. RAW events were defined as confirmed disability progression in patients who experienced at least 1 clinical relapse during the follow-up period.

Treatment discontinuation was defined on the specific characteristics of each DMT. For S1PRMs, discontinuation referred to any treatment interruption, with or without the initiation of a subsequent DMT, at any point during follow-up. For cladribine, discontinuation was defined as either (1) failure to complete the 2 planned treatment courses (year 1 and year 2) or (2) initiation of another DMT within 4 years of follow-up. Cox proportional hazards regression models, adjusted for visit frequency and stratified by matched pairs, were used to compare discontinuation risk between groups. Reasons for discontinuation were categorized as clinical and/or MRI activity, adverse events, or other reasons.

Given the exploratory study design, no correction for multiplicity was applied. Two-tailed *P* < .05 was considered statistically significant. Data were analyzed using SPSS Statistical software version 23.0 (IBM).

## Results

### Descriptive Analysis

**Figure 1.**
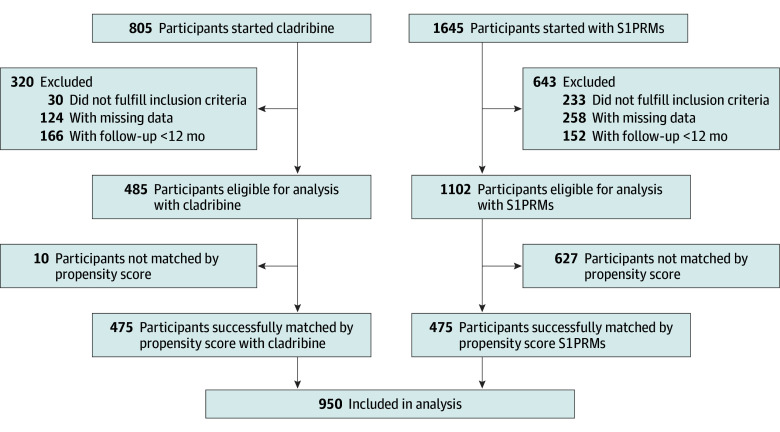
Patient Inclusion Flowchart for Analysis of Treatment-Naive Patients With Relapsing-Remitting Multiple Sclerosis Treated With Cladribine or Sphingosine-1-Phosphate Receptor Modulators (S1PRMs) With ≥12 Months of Follow-Up

Between January 2011 and October 2021, 2450 treatment-naive individuals with RRMS started treatment with cladribine (805 patients) or S1PRMs (1645 patients; fingolimod, 1430 patients; ozanimod, 157 patients; and ponesimod, 58 patients) across 108 MS centers in Italy. After excluding individuals not meeting eligibility criteria, with missing data, or less than 12 months of follow-up, 485 patients treated with cladribine and 1102 patients treated with S1PRM were retained for analysis (Figure 1).

The median (range) follow-up was 28 (12-98) months for cladribine and 64 (12-146) months for S1PRMs before matching. At baseline, patients treated with cladribine were younger, had shorter disease duration, lower EDSS scores, and more pretreatment relapses compared with those receiving the S1PRMs group (Table 1). Differences were no longer significant after PS matching, leaving 950 patients (475 per group; mean [SD] age, 34.7 [10.7] years; 686 female [72.2%]), with balanced covariates (Cohen *d* < 0.20; PS differences, 0.59 for the unmatched cohort and 0.02 for the matched cohort). Pairwise censoring yielded median (IQR) follow-up of 25 (12-60) months in both groups. Early relapses (ie, within 3 months of treatment initiation) occurred in 27 patients (5.7%) taking cladribine and 31 patients (6.5%) taking S1PRMs, while early disability worsening (ie, within 6 months) was observed in 9 patients (1.9%) taking cladribine vs 12 patients (2.5%) taking S1PRMs.

**Table 1.** Baseline Characteristics of Included Patients Before and After Matching

Characteristics	Unmatched cohort			Matched cohort		
	Patients, No. (%)		Cohen *d*	Patients, No. (%)		Cohen *d*
	Cladribine (n = 485)	S1PRMs (n = 1182)		Cladribine (n = 475)	S1PRMs (n = 475)	
Type of S1PRM therapy						
Fingolimod	NA	1033 (93.7)	NA	NA	406 (85.5)	NA
Ozanimod	NA	58 (5.3)	NA	NA	58 (12.2)	NA
Ponesimod	NA	11 (1.0)	NA	NA	11 (2.3)	NA
Sex						
Female	348 (71.8)	773 (62.9)	0.19	343 (71.2)	343 (71.2)	0
Male	137 (28.2)	409 (37.1)		137 (28.8)	137 (28.8)	
Age, mean (SD) y	34.3 (10.0)	37.4 (11.2)^a^	0.40	34.6 (10.1)	34.9 (11.3)	0.05
Location of clinical onset: optic nerve	110 (22.7)	222 (20.1)	0.06	97 (20.4)	97 (20.4)	0
Time since first symptom, mean (SD), y	2.6 (4.1)	4.2 (6.1)^a^	0.81	2.8 (4.6)	2.9 (5.0)	0.04
Expanded Disability Status Scale score, median (IQR)	2.0 (0.0-6.5)	2.0 (0.0-6.5)^a^	0.55	2.0 (0.0-6.5)	2.0 (0.0-6.5)	0.02
No. of pretreatment relapses, mean (SD)^b^	1.5 (1.2)	1.7 (1.5)	0.12	1.7 (1.1)	1.8 (1.3)	0.04
Pretreatment annualized relapse rate, mean (SD)	2.1 (2.4)	1.6 (2.2)^a^	0.35	2.4 (2.4)	2.3 (2.2)	0.07
Baseline gadolinium-enhancing lesions	242 (57.3)	284 (38.1)	0.38	233 (56.7)	138 (41.7)	0.30
Propensity score, mean (SD)^b^	0.337 (0.087)	0.292 (0.100)^a^	0.59	0.319 (0.084)	0.318 (0.083)	0.02
Follow-up length, median (IQR), mo^b^	28 (12-98)	64 (12-146)^a^	NA	28 (12-98)	64 (12-180)	NA
Visit frequency, mean (SD), No./y^b^	2.5 (1.7)	2.2 (1.6)^a^	NA	2.2 (1.5)	2.5 (1.6)	NA
Scan frequency, mean (SD), No./y^b^^,^^c^	1.1 (0.5)	0.8 (0.5)	NA	1.0 (0.3)	0.9 (0.3)	NA

Abbreviations: NA, not applicable; S1PRM, sphingosine-1-phosphate receptor modulators.

^a^ Significant difference at a 2-sided α-level <.05 by the Fisher exact (for categorical variables) and Mann-Whitney *U* (for continuous variables) tests in the unmatched cohort.

^b^ Not included in the propensity score estimation.

^c^ Estimated on patients’ subsamples with available magnetic resonance imaging data.

### Comparative Effectiveness Analyses

**Figure 2.**
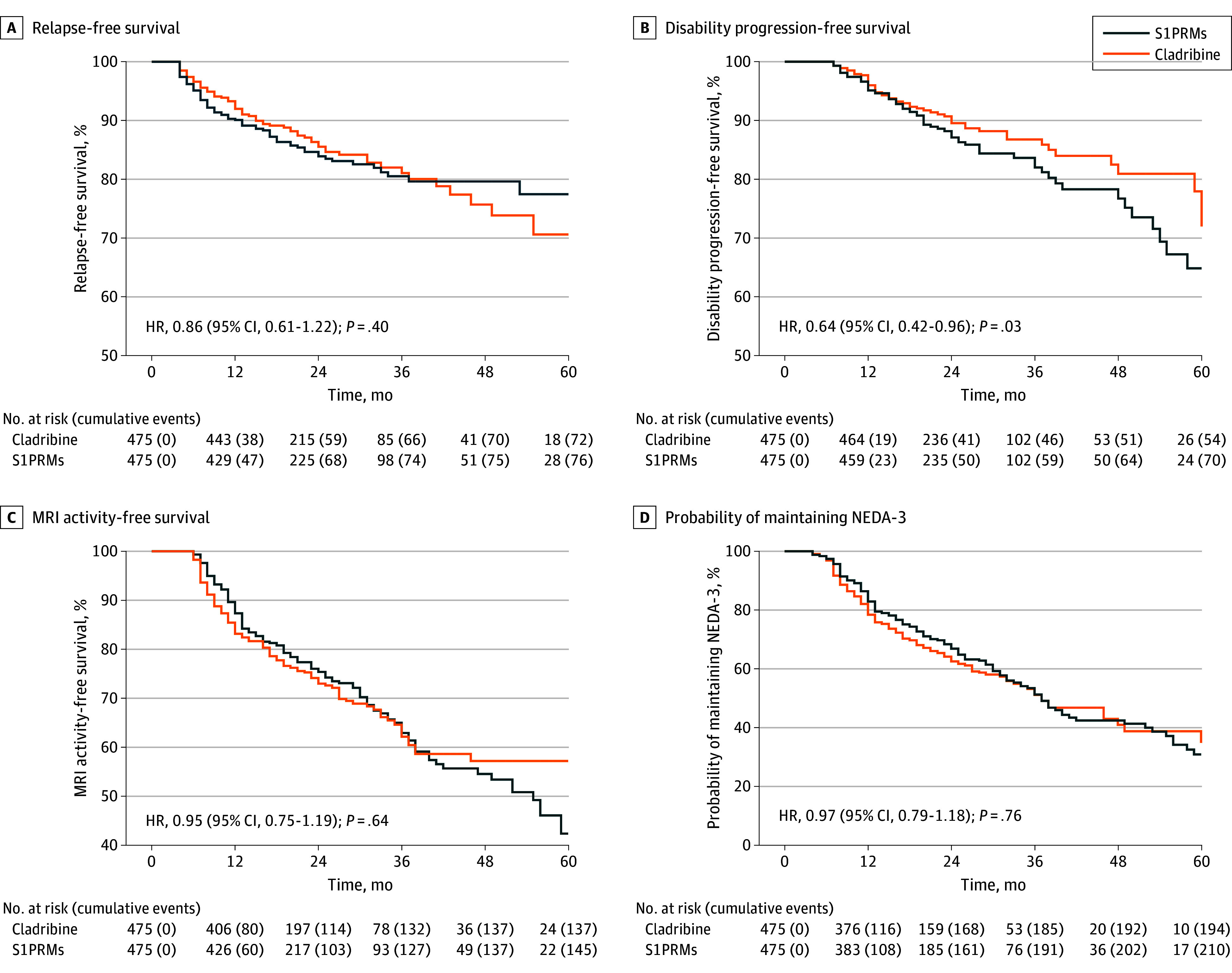
Kaplan-Meier Curves Comparing Patients Taking Cladribine vs Sphingosine-1-Phosphate Receptor Modulators (S1PRMs) Graphs show probability of relapse-free survival (A), disability progression–free survival (B), magnetic resonance imaging (MRI) activity (C), and (D) no evidence of disease activity with 3 components (NEDA-3) loss. HR indicates hazard ratio.

The primary analysis results (Table 2 and Figure 2) showed that 72 patients (15.2%) in the cladribine group and 76 patients (16.0%) in the S1PRMs group experienced relapses. There was no difference in relapse risk between the groups (hazard ratio [HR], 0.86; 95% CI, 0.61-1.22; *P* = .40). However, patients treated with cladribine showed a significantly lower risk of EDSS worsening than those receiving S1PRMs (HR, 0.64; 95% CI, 0.42-0.96; *P* = .03), with 54 patients (11.4%) vs 70 patients (14.7%) experiencing disability accrual. MRI activity was observed in 137 patients (31.3%) in the cladribine group and 145 patients (34.8%) in the S1PRM group. Loss of NEDA-3 status occurred in 194 patients (44.4%) in the cladribine group and 219 patients (52.5%) in the S1PRM group. There were no significant differences in the risk of MRI activity (HR, 0.95; 95% CI, 0.75-1.19; *P* = .64) or in the loss of NEDA-3 status (HR, 0.97; 95% CI, 0.79-1.18; *P* = .76).

**Table 2.** Primary and Secondary Analyses Report Outcome Rates and Adjusted Cox Models

Analyses and outcomes	Patients reaching outcome, No. (%)		HR (95% CI)^a^	*P* value
	Cladribine	S1PRMs		
Case base scenario				
Relapse	72 (15.2)	76 (16.0)	0.86 (0.61-1.22)	.40
EDSS worsening	54 (11.4)	70 (14.7)	0.64 (0.42-0.96)	.03
MRI activity^b^	137 (31.3)	145 (34.8)	0.95 (0.75-1.19)	.64
Loss of NEDA-3^b^	194 (44.4)	219 (52.5)	0.97 (0.79-1.18)	.76
Sensitivity analysis 1: restricted to patients aged <40 y				
Relapse	46 (18.6)	49 (19.8)	0.95 (0.61-1.47)	.81
EDSS worsening	23 (9.3)	37 (15.0)	0.52 (0.28-0.93)	.03
MRI activity^b^	81 (38.9)	85 (40.8)	0.96 (0.66-1.40)	.84
Loss of NEDA-3^b^	103 (49.5)	107 (51.4)	0.92 (0.65-1.31)	.64
Sensitivity analysis 2: restricted to diagnoses based on 2017 revised McDonald Criteria				
Relapse	18 (9.5)	24 (12.6)	0.56 (0.26-1.20)	.14
EDSS worsening	15 (7.9)	31 (16.3)	0.48 (0.25-0.93)	.03
MRI activity^b^	54 (33.7)	58 (36.2)	0.90 (0.69-1.25)	.65
Loss of NEDA-3^b^	67 (41.9)	66 (41.2)	0.94 (0.62-1.41)	.76
Sensitivity analysis 3: restricted to patients scoring ≤3.0 at EDSS				
Relapse	66 (16.6)	70 (17.6)	0.76 (0.52-1.10)	.15
EDSS worsening	50 (12.6)	65 (16.4)	0.62 (0.39-0.98)	.04
MRI activity^b^	115 (32.2)	124 (34.7)	0.93 (0.68-1.26)	.63
Loss of NEDA-3^b^	156 (43.7)	166 (46.5)	0.89 (0.67-1.17)	.40
Sensitivity analysis 4: restricted to patients taking fingolimod vs their counterparts taking cladribine^c^				
Relapse	68 (16.7)	73 (18.0)	0.82 (0.57-1.18)	.30
EDSS worsening	52 (12.8)	68 (16.7)	0.65 (0.42-0.99)	.04
MRI activity^b^	130 (35.0)	134 (36.2)	1.00 (0.80-1.42)	.58
Loss of NEDA-3^b^	190 (51.3)	207 (55.9)	0.97 (0.76-1.24)	.81
Sensitivity analysis 5: restricted to patients with available baseline gadolinium-enhancing lesion data^d^				
Relapse	54 (15.0)	64 (16.8)	0.72 (0.46-1.12)	.14
EDSS worsening	35 (10.8)	55 (14.4)	0.67 (0.45-1.00)	.05
MRI activity^b^	115 (35.4)	122 (37.5)	0.79 (0.59-1.07)	.13
Loss of NEDA-3^b^	148 (45.5)	157 (48.3)	0.78 (0.58-1.02)	.07
Sensitivity analysis 6: restricted to patients with follow-up ≥36 mo				
Relapse	27 (22.9)	16 (13.6)	1.81 (1.02-3.20)	.04
EDSS worsening	17 (14.4)	23 (19.5)	0.78 (0.41-1.47)	.44
MRI activity^b^	30 (42.8)	24 (34.3)	1.68 (0.87-3.28)	.12
Loss of NEDA-3^b^	47 (67.1)	35 (50.0)	2.08 (1.18-3.67)	.01

Abbreviations: EDSS, expanded disability status scale; HR, hazard ratio; MRI, magnetic resonance imaging; NEDA-3, no evidence of disease activity; S1PRMs, sphingosine-1-phosphate receptor modulators.

^a^ HRs less than 1.0 favor cladribine, and HRs greater than 1.0 favor S1PRMs.

^b^ MRI data were not available for 96 patients (38 in cladribine group and 58 in S1PRMs group).

^c^ Sixty-nine patients who were treated with S1PRMs other than fingolimod (ozanimod and ponesimod) were excluded.

^d^ Analysis was conducted on 382 patient pairs after propensity score matching and pairwise procedures were rerun on 1167 patients (422 in the cladribine group and 745 in the S1PRM group) for whom baseline gadolinium-enhancing lesion data was available.

A lower number of matched pairs was obtained for MRI-related outcomes due to missing data for 96 patients (38 in the cladribine group and 58 in the S1PRM group). Baseline characteristics did not differ between these 96 patients and the remaining 854 patients (eTable 2 in Supplement 1).

### Sensitivity Analyses

In analysis 1 (494 patients; 247 pairs), restricted to patients younger than 40 years, cladribine was associated with a significantly lower risk of EDSS worsening (HR, 0.52; 95% CI, 0.28-0.93; *P* = .03), with no significant differences in relapse (HR, 0.95; 95% CI, 0.61-1.47; *P* = .81), MRI activity (HR, 0.96; 95% CI, 0.66-1.40; *P* = .84), or NEDA-3 loss (HR, 0.92; 95% CI, 0.65-1.31; *P* = .64). In analysis 2 (380 patients; 190 pairs), limited to patients whose diagnoses were made according to the 2017 McDonald Criteria, cladribine again showed a significantly lower risk of EDSS worsening (HR, 0.48; 95% CI, 0.25-0.93; *P* = .03), with nonsignificant differences in relapse (HR, 0.56; 95% CI, 0.26-1.20; *P* = .14), MRI activity (HR, 0.90; 95% CI, 0.69-1.25; *P* = .65), and NEDA-3 loss (HR, 0.94; 95% CI, 0.62-1.41; *P* = .76). In analysis 3 (794 patients; 397 pairs), restricted to patients with baseline EDSS less than or equal to 3.0, cladribine remained significantly protective against disability progression (HR, 0.62; 95% CI, 0.39-0.98; *P* = .04), while differences in relapse (HR, 0.76; 95% CI, 0.52-1.10; *P* = .15), MRI activity (HR, 0.93; 95% CI, 0.68-1.26; *P* = .63), and NEDA-3 loss (HR, 0.89; 95% CI, 0.67-1.17; *P* = .40) were again not significant. In analysis 4 (812 patients; 406 pairs), which was limited to patients treated with fingolimod compared with those treated with cladribine, the latter group again demonstrated a significantly lower risk of EDSS deterioration (HR, 0.65; 95% CI, 0.42-0.99; *P* = .04). No significant differences emerged for relapse risk (HR, 0.82; 95% CI, 0.57-1.18; *P* = .30), MRI activity (HR, 1.00; 95% CI, 0.80-1.42; *P* = .58), or NEDA-3 loss (HR, 0.97; 95% CI, 0.76-1.24; *P* = .81). Analysis 5 (764 patients; 382 pairs) was conducted after the PS-matching and pairwise procedures were rerun for 1167 patients (422 in the cladribine group and 745 in the S1PRMs group) for whom baseline gadolinium-enhancing lesion data were available. This analysis found that for cladribine, the HR for EDSS worsening decreased but was not statistically significant (HR, 0.67; 95% CI, 0.45-1.00; *P* = .05), while the risk of relapse (HR, 0.72; 95% CI, 0.46-1.12; *P* = .14), MRI activity (HR, 0.79; 95% CI, 0.59-1.07; *P* = .13), and NEDA-3 loss (HR, 0.78; 95% CI, 0.58-1.02; *P* = .07) did not differ significantly between the 2 groups. In contrast, analysis 6 (236 patients; 118 pairs), which was limited to patients with more than 36 months of follow-up, revealed a significantly higher relapse risk (HR, 1.81; 95% CI, 1.02-3.20; *P* = .04) and increased NEDA-3 loss (HR, 2.08; 95% CI, 1.18-3.67; *P* = .01) in the cladribine group, with no significant differences in EDSS worsening (HR, 0.78; 95% CI, 0.41-1.47; *P* = .44) or MRI activity (HR, 1.68; 95% CI, 0.87-3.28; *P* = .12). Table 2 summarizes these findings.

### PIRA and RAW Analysis

In the PIRA and RAW analysis, we found that a lower number of patients treated with cladribine experienced PIRA events (28 patients) than those treated with S1PRMs (42 patients; HR, 0.40; 95% CI, 0.20-0.79; *P* = .009). However, no significant difference in RAW events was found between the cladribine and S1PRM groups (26 vs 28 patients; HR, 0.58; 95% CI, 0.13-2.58; *P* = .48). Due to the limited availability of MRI data, we were unable to assess progression independent of both relapse and MRI activity.

### Risk Variables

None of the baseline variables, including sex, age, location of onset (optic nerve vs others), time since first symptom, or EDSS score, was significantly associated with the risk of reaching any predefined outcomes in either treatment group. Older age and male sex had reduced HRs for certain outcomes but these were not statistically significant (eTable 3 in Supplement 1).

### Treatment Discontinuation

Treatment discontinuation occurred in 86 patients (18.1%) treated with cladribine and 102 patients (20.5%) taking S1PRMs (HR, 0.92; 95% CI, 0.67-1.15; *P* = .58). Discontinuation due to clinical and/or MRI activity or adverse effects was more frequent with S1PRMs (eTable 4 in Supplement 1). A total of 188 patients switched therapy, with 96 (51.1%) undergoing escalation to monoclonal antibodies and 44 (23.4%) deescalation to moderate-efficacy DMTs with no between-group difference. Data on subsequent DMT were missing for 37 patients discontinuing S1PRMs (eTable 5 in Supplement 1).

## Discussion

This comparative effectiveness research study provides comparative evidence on the effectiveness of 2 oral treatments, cladribine and S1PRMs, in treatment-naive patients with RRMS. In the unmatched cohort, the S1PRM group was older, with longer disease duration, lower ARR, and fewer gadolinium-enhancing lesions than the cladribine group—differences likely reflecting distinct prescribing patterns influenced by the staggered availability of these drugs. Baseline characteristics in the PS–matched cohort broadly aligned with the Italian eligibility criteria for highly active, treatment-naïve patients. Our findings indicate that both therapies achieved similar short-term NEDA-3 outcomes, but cladribine was associated with a significantly lower risk of disability progression over a median follow-up of 25 months. This effect was confirmed in sensitivity analyses of patients younger than 40 years, with baseline EDSS less than or equal to 3, whose diagnoses were made per the 2017 McDonald criteria or treated only with fingolimod. No significant differences were found in relapse rates or MRI activity between the groups.

To date, no head-to-head randomized clinical trials have directly compared cladribine with any S1PRMs. Nevertheless, contextualizing our results alongside those from pivotal phase 3 trials provides meaningful insights. In our cohort, 44.4% of patients treated with cladribine lost NEDA-3 status at 25 months. By comparison, in the CLARITY trial, 56% of treatment-naive participants who received the 3.5 mg/kg cladribine regiment lost NEDA-3 at 96 weeks,^40^ reflecting modest differences likely due to variations in treatment dynamics, patient selection, or clinical management. For S1PRMs, our findings align with the FREEDOMS trial,^14^ in which 84% of treatment-naive patients with RRMS receiving fingolimod 0.5 mg remained free from confirmed disability progression at 2 years, increasing to 84.7% in those with rapidly evolving severe RRMS.^41^ In our cohort, 87% of patients treated with S1PRMs showed no evidence of disability worsening at the 2-year follow-up. An indirect network meta-analysis on 6 randomized clinical trials and conducted using a bayesian and Markov chain Monte Carlo approach found no significant difference in achieving NEDA-3 between cladribine tablets and fingolimod over 24 months. Limited data precluded direct comparison of clinical NEDA, but cladribine demonstrated superior MRI-based NEDA outcomes.^42^

Our study is unique in focusing exclusively on treatment-naive patients with RRMS, allowing a cleaner comparison of cladribine and S1PRMs without the confounding influence of prior therapies. Only 1 other study has specifically addressed this population, comparing a CLARITY trial subgroup with a fingolimod observational cohort, and found no significant differences in relapses or disability, although limited power from small sample size may have reduced the statistical power of the analysis.^31^

Most other comparative studies included heterogeneous, previously treated patients, yielding conflicting results. A PS-matched analysis from MSBase showed similar efficacy over 1 year, but greater disability improvement with cladribine.^33^ The MERLYN study reported comparable ARR and lower discontinuation rates with cladribine at 12 months,^32^ although its mixed cohort and reliance on descriptive statistics limit interpretation. Conversely, a recent MSBase-UK study found lower ARR with cladribine but no difference in disability.^29^ Despite similar follow-up (approximately 2.1 years), results may reflect that their population was more likely to switch medications, highlighting how prior treatment exposure complicates comparisons.

In our cohort, the reduced risk of disability worsening with cladribine was mainly associated with a significant reduction in PIRA events, whereas RAW did not differ between groups. This finding suggests that cladribine may effectively mitigate subclinical disease progression independently of overt inflammation. These findings are consistent with those from the MAGNIFY-MS study, which reported reductions in both PIRA and RAW over 2 years in patients treated with cladribine with highly active disease.^43^ Treatment-naive individuals demonstrated more favorable outcomes, with 24-month freedom from composite PIRA of 89.0%, vs 81.9% in treatment-experienced patients, and freedom from composite confirmed disability accumulation at 89.0% vs 77.9%, supporting early cladribine initiation.^43^ In our study, PIRA was identified using a simplified, indirect method due to variable follow-up, precluding strict adherence to standardized definitions and requiring rebaselining and confirmation at fixed intervals.^44,45^ Despite this exploratory approach, uniform application across groups permits valid comparative assessment within observational constraints.

Another key observation was that, beyond 36 months, cladribine was associated with higher risk of relapse and loss of NEDA-3 status. This novel finding requires cautious interpretation. NEDA-3, although widely adopted, is sensitive to minimal activity and does not reflect the severity or impact of events; it should, therefore, be complemented by other measures of therapeutic effectiveness. For instance, treatment discontinuation rates remained comparable, stabilizing at approximately 20% in both groups. Moreover, the increased disease activity with cladribine was statistically significant but based on roughly one-quarter of the cohort, requiring confirmation with longer follow-up. Our results align with the CLARITY trial and its 4-year extension, in which 75.6% of patients treated with cladribine 3.5 mg/kg for 2 years and then switched to placebo remained relapse-free, and 72.4% had no confirmed EDSS progression.^46^ These rates were lower than in patients who continued treatment for an additional 2 years, and MRI activity tended to re-emerge, particularly when treatment phases were separated by long gaps.^47^ This supports the notion that discontinuation reduces disease control.

Overall, our data, together with prior evidence, suggest that cladribine’s long-term effectiveness may decline due to its intermittent dosing schedule, based on 2 short treatment courses followed by extended drug-free intervals. In contrast, S1PRMs, administered continuously, provided more stable disease control in our cohort beyond 36 months, with lower risk of reactivation.

Our analysis did not identify any baseline demographic or clinical variables as factors significantly associated with treatment response or disease activity in either group. A trend toward lower relapse risk and reduced loss of NEDA-3 was observed in older patients, but this did not reach statistical significance. Similarly, trends for sex and other factors lacked statistical support and offered no clear guidance for personalized treatment selection.

### Strengths and Limitations

The main strength of this study is its large sample size and rigorous PS-matching, which minimized confounding and enhanced comparability between groups; sensitivity analyses further supported robustness. Limitations include its observational design, with nonrandomized treatment allocation and potential residual confounding despite matching. The median 25-month follow-up may be insufficient to capture long-term effects, particularly given different dosing schedules; a sensitivity analysis of patients with follow-up for 36 months or longer partly addressed this. Selection bias may persist, as a larger proportion of patients treated with S1PRM were excluded during matching. Adherence and tolerability were not assessed but may have influenced outcomes. Data collection inconsistencies, especially in timing and frequency of clinical visits and MRI, could affect accuracy. No primary outcome was prespecified, and no adjustment for multiple comparisons was applied, so findings should be considered exploratory. Furthermore, grouping the 3 S1PRMs assumes similar effectiveness, although differential outcomes cannot be excluded.

## Conclusions

In this comparative effectiveness study of treatment-naive patients with RRMS, cladribine was associated with greater benefit in delaying disability progression, particularly among younger patients and those with lower baseline EDSS, largely driven by reduced PIRA events. Both therapies showed similar efficacy in controlling relapses and MRI activity initially, but from year 3 onward cladribine was associated with higher risk of relapse and MRI reactivation. This attenuation suggests that earlier redosing or timely treatment switching may be required for sustained control. Future prospective head-to-head studies would ideally confirm these findings, although practical challenges emphasize the need for validation in other long-term observational cohorts. Our dataset could also be re-examined as follow-up extends. Evaluating effects on quality of life, cognition, and adherence and identifying biomarkers associated with treatment response will further inform individualized treatment strategies in MS.

## Notes

Supplement 1. eTable 1. Availability of Baseline MRI Data in the Patients Eligible for Analysis (n = 1587) eTable 2. Comparison of Baseline Characteristics Between Patients With and Without Follow-up MRI Data in the PS-Matched Sample eTable 3. Association Between Baseline Characteristics and Response to Cladribine and S1PRMs eTable 4. Reasons for Treatment Suspension with Cladribine or S1PRMs eTable 5. Subsequent Therapies Following Discontinuation of Cladribine or S1PRMs

Supplement 2. Data Sharing Statement

## References

[1] Makhani N, Tremlett H. The multiple sclerosis prodrome. Nat Rev Neurol. 2021;17(8):515-521. doi:10.1038/s41582-021-00519-3 34155379 PMC8324569

[2] Stankiewicz JM, Weiner HL. An argument for broad use of high efficacy treatments in early multiple sclerosis. Neurol Neuroimmunol Neuroinflamm. 2019;7(1):e636. doi:10.1212/NXI.0000000000000636 31757815 PMC6935832

[3] Harding K, Williams O, Willis M, . Clinical outcomes of escalation vs early intensive disease-modifying therapy in patients with multiple sclerosis. JAMA Neurol. 2019;76(5):536-541. doi:10.1001/jamaneurol.2018.4905 30776055 PMC6515582

[4] Prosperini L, Mancinelli CR, Solaro CM, . Induction versus escalation in multiple sclerosis: a 10-year real world study. Neurotherapeutics. 2020;17(3):994-1004. doi:10.1007/s13311-020-00847-0 32236822 PMC7609676

[5] Karampampa K, Gyllensten H, Murley C, . Early vs. late treatment initiation in multiple sclerosis and its impact on cost of illness: a register-based prospective cohort study in Sweden. Mult Scler J Exp Transl Clin. Published on April 24, 2022;8(2):20552173221092411. doi:10.1177/20552173221092411 35496759 PMC9044795

[6] Filippi M, Amato MP, Centonze D, . Early use of high-efficacy disease-modifying therapies makes the difference in people with multiple sclerosis: an expert opinion. J Neurol. 2022;269(10):5382-5394. doi:10.1007/s00415-022-11193-w 35608658 PMC9489547

[7] Freeman L, Longbrake EE, Coyle PK, Hendin B, Vollmer T. High-efficacy therapies for treatment-naïve individuals with relapsing-remitting multiple sclerosis. CNS Drugs. 2022;36(12):1285-1299. doi:10.1007/s40263-022-00965-7 36350491 PMC9645316

[8] Toscano S, Spelman T, Ozakbas S, ; MSBase Study Group. First-year treatment response predicts the following 5-year disease course in patients with relapsing-remitting multiple sclerosis. Neurotherapeutics. 2025;22(2):e00552. doi:10.1016/j.neurot.2025.e00552 39965993 PMC12014414

[9] Buron MD, Chalmer TA, Sellebjerg F, . Initial high-efficacy disease-modifying therapy in multiple sclerosis: a nationwide cohort study. Neurology. 2020;95(8):e1041-e1051. doi:10.1212/WNL.0000000000010135 32636328

[10] Wiendl H, Gold R, Berger T, ; Multiple Sclerosis Therapy Consensus Group. Multiple Sclerosis Therapy Consensus Group (MSTCG): position statement on disease-modifying therapies for multiple sclerosis (white paper). Ther Adv Neurol Disord. Published online August 18, 2021;14:17562864211039648. doi:10.1177/17562864211039648 34422112 PMC8377320

[11] Hrnciarova T, Drahota J, Spelman T, . Does initial high efficacy therapy in multiple sclerosis surpass escalation treatment strategy? a comparison of patients with relapsing-remitting multiple sclerosis in the Czech and Swedish national multiple sclerosis registries. Mult Scler Relat Disord. 2023;76:104803. doi:10.1016/j.msard.2023.104803 37329786

[12] Rotstein D, Montalban X. Reaching an evidence-based prognosis for personalized treatment of multiple sclerosis. Nat Rev Neurol. 2019;15(5):287-300. doi:10.1038/s41582-019-0170-8 30940920

[13] Filippi M, Danesi R, Derfuss T, . Early and unrestricted access to high-efficacy disease-modifying therapies: a consensus to optimize benefits for people living with multiple sclerosis. J Neurol. 2022;269(3):1670-1677. doi:10.1007/s00415-021-10836-8 34626224 PMC8501364

[14] Kappos L, Radue EW, O’Connor P, ; FREEDOMS Study Group. A placebo-controlled trial of oral fingolimod in relapsing multiple sclerosis. N Engl J Med. 2010;362(5):387-401. doi:10.1056/NEJMoa0909494 20089952

[15] Comi G, Kappos L, Selmaj KW, ; SUNBEAM Study Investigators. Safety and efficacy of ozanimod versus interferon beta-1a in relapsing multiple sclerosis (SUNBEAM): a multicentre, randomised, minimum 12-month, phase 3 trial. Lancet Neurol. 2019;18(11):1009-1020. doi:10.1016/S1474-4422(19)30239-X 31492651

[16] Kappos L, Fox RJ, Burcklen M, . Ponesimod compared with teriflunomide in patients with relapsing multiple sclerosis in the active-comparator phase 3 OPTIMUM study: a randomized clinical trial. JAMA Neurol. 2021;78(5):558-567. doi:10.1001/jamaneurol.2021.0405 33779698 PMC8008435

[17] Giovannoni G, Comi G, Cook S, ; CLARITY Study Group. A placebo-controlled trial of oral cladribine for relapsing multiple sclerosis. N Engl J Med. 2010;362(5):416-426. doi:10.1056/NEJMoa0902533 20089960

[18] Leist TP, Comi G, Cree BA, ; Oral Cladribine for Early MS (ORACLE MS) Study Group. Effect of oral cladribine on time to conversion to clinically definite multiple sclerosis in patients with a first demyelinating event (ORACLE MS): a phase 3 randomised trial. Lancet Neurol. 2014;13(3):257-267. doi:10.1016/S1474-4422(14)70005-5 24502830

[19] Arena S, Chisari CG, Toscano S, . Real-world effectiveness of cladribine for patients with multiple sclerosis: a Sicilian multicentric experience (rewind study). Curr Neuropharmacol. 2024;22(7):1271-1283. doi:10.2174/1570159X21666230322140711 36946484 PMC10964096

[20] Petracca M, Ruggieri S, Barbuti E, . Predictors of cladribine effectiveness and safety in multiple sclerosis: a real-world, multicenter, 2-year follow-up study. Neurol Ther. 2022;11(3):1193-1208. doi:10.1007/s40120-022-00364-6 35653061 PMC9338179

[21] Zanetta C, Rocca MA, Meani A, . Effectiveness and safety profile of cladribine in an Italian real-life cohort of relapsing-remitting multiple sclerosis patients: a monocentric longitudinal observational study. J Neurol. 2023;270(7):3553-3564. doi:10.1007/s00415-023-11700-7 37027018 PMC10080181

[22] Annovazzi P, Frau J, Margoni M, . Two year relapse-free and NEDA status with Cladribine in a real life population: a multicentre study (poster presentation P842). Poster presented at the 37th European Committee for Treatment and Research in Multiple Sclerosis; October 13–15, 2021.

[23] Rauma I, Viitala M, Kuusisto H, . Finnish multiple sclerosis patients treated with cladribine tablets: a nationwide registry study. Mult Scler Relat Disord. 2022;61:103755. doi:10.1016/j.msard.2022.103755 35483129

[24] Potuznik P, Drahota J, Horakova D, . Real-world effectiveness of cladribine as an escalation strategy for MS: Insights from the Czech nationwide ReMuS registry. J Cent Nerv Syst Dis. Published online July 24, 2024;16:11795735241262743. doi:10.1177/11795735241262743 39055049 PMC11271105

[25] Manni A, Oggiano F, Palazzo C, . Clinical and biological predictors of cladribine effectiveness in multiple sclerosis: a real-world, single centre study considering a two-year interval from year-2 dosing. J Neurol Sci. 2024;462:123070. doi:10.1016/j.jns.2024.123070 38850773

[26] Cannizzaro M, Ferré L, Clarelli F, . Early use of fingolimod is associated with better clinical outcomes in relapsing-remitting multiple sclerosis patients. J Neurol. 2022;269(10):5596-5605. doi:10.1007/s00415-022-11227-3 35759013

[27] Hohlfeld R, Kappos L, Tomic D, Early fingolimod treatment improves disease outcomes at 2 and 4 years in patients with relapsing-remitting multiple sclerosis. Neurology. 2017;88(16 Suppl):6, 332. doi:10.1212/WNL.88.16_supplement.P6.3

[28] Brownlee W, Amin A, Ashton L, Herbert A. Real-world use of cladribine tablets (completion rates and treatment persistence) in patients with multiple sclerosis in England: The CLARENCE study. Mult Scler Relat Disord. 2023;79:104951. doi:10.1016/j.msard.2023.104951 37639781

[29] Roos I, Sharmin S, Malpas C, . Effectiveness of cladribine compared to fingolimod, natalizumab, ocrelizumab and alemtuzumab in relapsing-remitting multiple sclerosis. Mult Scler. 2024;30(9):1163-1175. doi:10.1177/13524585241267211 39087208

[30] Spelman T, Ozakbas S, Alroughani R, . Comparative effectiveness of cladribine tablets versus other oral disease-modifying treatments for multiple sclerosis: results from MSBase registry. Mult Scler. 2023;29(2):221-235. doi:10.1177/13524585221137502 36433775 PMC9925904

[31] Signori A, Saccà F, Lanzillo R, . Cladribine vs other drugs in MS: merging randomized trial with real-life data. Neurol Neuroimmunol Neuroinflamm. 2020;7(6):e878. doi:10.1212/NXI.0000000000000878 32801167 PMC7641098

[32] Brownlee WJ, Haghikia A, Hayward B, . Comparative effectiveness of cladribine tablets versus fingolimod in the treatment of highly active multiple sclerosis: a real-world study. Mult Scler Relat Disord. 2023;76:104791. doi:10.1016/j.msard.2023.104791 37343465

[33] Kalincik T, Jokubaitis V, Spelman T, ; MSBase Study Group. Cladribine versus fingolimod, natalizumab and interferon β for multiple sclerosis. Mult Scler. 2018;24(12):1617-1626. doi:10.1177/1352458517728812 28857680

[34] Trojano M, Bergamaschi R, Amato MP, ; Italian Multiple Sclerosis Register Centers Group. The Italian multiple sclerosis register. Neurol Sci. 2019;40(1):155-165. doi:10.1007/s10072-018-3610-0 30426289 PMC6329744

[35] McDonald WI, Compston A, Edan G, . Recommended diagnostic criteria for multiple sclerosis: guidelines from the International Panel on the diagnosis of multiple sclerosis. Ann Neurol. 2001;50(1):121-127. doi:10.1002/ana.1032 11456302

[36] Degenhardt A, Ramagopalan SV, Scalfari A, Ebers GC. Clinical prognostic factors in multiple sclerosis: a natural history review. Nat Rev Neurol. 2009;5(12):672-682. doi:10.1038/nrneurol.2009.178 19953117

[37] Thompson AJ, Banwell BL, Barkhof F, . Diagnosis of multiple sclerosis: 2017 revisions of the McDonald criteria. Lancet Neurol. 2018;17(2):162-173. doi:10.1016/S1474-4422(17)30470-2 29275977

[38] Leray E, Yaouanq J, Le Page E, . Evidence for a two-stage disability progression in multiple sclerosis. Brain. 2010;133(Pt 7):1900-1913. doi:10.1093/brain/awq076 20423930 PMC2892936

[39] Weideman AM, Tapia-Maltos MA, Johnson K, Greenwood M, Bielekova B. Meta-analysis of the age-dependent efficacy of multiple sclerosis treatments. Front Neurol. 2017;8:577. doi:10.3389/fneur.2017.00577 29176956 PMC5686062

[40] Giovannoni G, Cook S, Rammohan K, ; CLARITY study group. Sustained disease-activity-free status in patients with relapsing-remitting multiple sclerosis treated with cladribine tablets in the CLARITY study: a post-hoc and subgroup analysis. Lancet Neurol. 2011;10(4):329-337. doi:10.1016/S1474-4422(11)70023-0 21397565

[41] Devonshire V, Havrdova E, Radue EW, ; FREEDOMS study group. Relapse and disability outcomes in patients with multiple sclerosis treated with fingolimod: subgroup analyses of the double-blind, randomised, placebo-controlled FREEDOMS study. Lancet Neurol. 2012;11(5):420-428. doi:10.1016/S1474-4422(12)70056-X 22494956

[42] Bartosik-Psujek H, Kaczyński Ł, Górecka M, . Cladribine tablets versus other disease-modifying oral drugs in achieving no evidence of disease activity (NEDA) in multiple sclerosis-A systematic review and network meta-analysis. Mult Scler Relat Disord. 2021;49:102769. doi:10.1016/j.msard.2021.102769 33516133

[43] De Stefano N, Wiendl H, Barkhof F, . Low rate of progression independent of relapse activity (PIRA) in patients with relapsing multiple sclerosis treated with cladribine tablets. Mult Scler Relat Disord. 2024;92:106121. doi:10.1016/j.msard.2024.106121

[44] Müller J, Cagol A, Lorscheider J, . Harmonizing definitions for progression independent of relapse activity in multiple sclerosis: a systematic review. JAMA Neurol. 2023;80(11):1232-1245. doi:10.1001/jamaneurol.2023.3331 37782515

[45] Prosperini L, Ruggieri S, Haggiag S, Tortorella C, Pozzilli C, Gasperini C. Prognostic accuracy of NEDA-3 in long-term outcomes of multiple sclerosis. Neurol Neuroimmunol Neuroinflamm. 2021;8(6):e1059. doi:10.1212/NXI.0000000000001059 34373345 PMC8353667

[46] Giovannoni G, Soelberg Sorensen P, Cook S, . Safety and efficacy of cladribine tablets in patients with relapsing-remitting multiple sclerosis: results from the randomized extension trial of the CLARITY study. Mult Scler. 2018;24(12):1594-1604. doi:10.1177/1352458517727603 28870107

[47] Comi G, Cook S, Rammohan K, . Long-term effects of cladribine tablets on MRI activity outcomes in patients with relapsing-remitting multiple sclerosis: the CLARITY Extension study. Ther Adv Neurol Disord. January 23, 2018;11:1756285617753365. doi:10.1177/1756285617753365 29399054 PMC5788142

[48] The Italian Multiple Sclerosis and Related Disorders Register. Italian Multiple Sclerosis Register. Accessed September 24, 2025. https://registroitalianosm.it/en/index.php

