# Error in Figure 2

**DOI:** 10.1001/jamanetworkopen.2025.52120

**Published:** 2025-12-15

In the Original Investigation titled “Comparative Effectiveness of Cladribine and S1P Receptor Modulators in Treatment-Naive Relapsing-Remitting MS,”^1^ published November 3, 2025, incorrect hazard ratios and *P *values were reported in panels A, C, and D in Figure 2. This article has been corrected.^1^
