# Supplementary material for: Comparative Effectiveness of Cladribine and S1P Receptor Modulators in Treatment-Naive Relapsing-Remitting MS
Source: JAMA Netw Open. 2025 Nov 3;8(11):e2541025. doi: 10.1001/jamanetworkopen.2025.41025 (PMC12584038; doi:10.1001/jamanetworkopen.2025.41025)
Supplement: Supplement 1. — eTable 1. Availability of Baseline MRI Data in the Patients Eligible for Analysis (n = 1587) eTable 2. Comparison of Baseline Characteristics Between Patients With and Without Follow-up MRI Data in the PS-Matched Sample eTable 3. Association Between Baseline Characteristics and Response to Cladribine and S1PRMs eTable 4. Reasons for Treatment Suspension with Cladribine or S1PRMs eTable 5. Subsequent Therapies Following Discontinuation of Cladribine or S1PRMs [file jamanetwopen-e2541025-s001.pdf]

Supplemental Online Content

Haggiag S, Prosperini L, Filippi M, et al. Comparative effectiveness of cladribine and S1P receptor modulators in treatment-naive relapse remitting MS. *JAMA Netw Open.* 2025;8(11):e2541025. doi:10.1001/jamanetworkopen.2025.41025

eTable 1. Availability of Baseline MRI Data in the Patients Eligible for Analysis (n = 1587)

eTable 2. Comparison of Baseline Characteristics Between Patients With and Without Follow-up MRI Data in the PS-Matched Sample

eTable 3. Association Between Baseline Characteristics and Response to Cladribine and S1PRMs

eTable 4. Reasons for Treatment Suspension with Cladribine or S1PRMs

eTable 5. Subsequent Therapies Following Discontinuation of Cladribine or S1PRMs

This supplemental material has been provided by the authors to give readers additional information about their work.

© 2025 Haggiag S et al. *JAMA Netw Open*.

eTABLE 1. Availability of Baseline MRI Data in the Patients Eligible for Analysis (n = 1587).

	Cladribine	S1PRMs
n	485	1102
Absence of GD+ lesions*	180 (37.1)	561 (41.8)
Presence of GD+ lesions*	242 (49.9)	284 (25.8)
Missing values*	63 (13.0)	357 (32.4)

Data are reported as n (%)

**p<0.001 by the Fisher's exact test*

© 2025 Haggiag S et al. *JAMA Netw Open*.

eTABLE 2. Comparison of Baseline Characteristics Between Patients With and Without Follow-up MRI Data in the PS-Matched Sample

	Available MRI data	Unavailable MRI data	*p*-value *
n	854	96	
Male sex, n (%)	244 (28.6)	30 (31.2)	0.632
Age, years	34.6 (10.1)	36.2 (10.8)	0.150
Topography of clinical onset: optic nerve, n (%)	174 (20.4)	20 (20.8)	0.888
Time since first symptom, years	2.9 (4.9)	2.8 (3.8)	0.775
EDSS score, median [interval]	1.8 (1.1)	1.9 (1.3)	0.692
No. of pre-treatment relapses	1.6 (1.4)	1.5 (1.4)	0.514
Pre-treatment annualised relapse rate	1.8 (2.4)	1.5 (1.7)	0.105

All values are mean (standard deviation) unless indicated otherwise

* by the Student's t-test for independent samples or Fisher's exact test, as appropriate for continuous or dichotomous variables, respectively.

© 2025 Haggiag S et al. *JAMA Netw Open*.

eTABLE 3. Association Between Baseline Characteristics and Response to Cladribine and S1PRMs

		Relapse	EDSS worsening	MRI activit[illegible]
		HR (95% CIs)	HR (95% CIs)	HR (95% CI[illegible]
Sex (male *vs.* female)	**Cladribine**	0.57 (0.31 to 1.03)	0.74 (0.39 to 1.40)	0.87 (0.60 to 1[illegible]
	**S1PRMs**	0.55 (0.30 to 1.00)	1.15 (0.70 to 1.91)	0.72 (0.48 to 1[illegible]
Age (each year)	**Cladribine**	0.98 (0.95 to 1.00)	1.02 (0.99 to 1.05)	0.98 (0.96 to 1[illegible]
	**S1PRMs**	0.98 (0.95 to 1.00)	1.01 (0.99 to 1.04)	0.98 (0.96 to 1[illegible]
Topography of clinical onset: optic nerve *vs.* other	**Cladribine**	1.35 (0.77 to 2.36)	0.64 (0.27 to 1.52)	1.11 (0.72 to 1[illegible]
	**S1PRMs**	1.14 (0.64 to 2.02)	0.59 (0.28 to 1.23)	1.28 (0.84 to 1[illegible]
Time since first symptom (each year)	**Cladribine**	1.00 (0.94 to 1.07)	1.00 (0.94 to 1.07)	0.95 (0.90 to 1[illegible]
	**S1PRMs**	0.89 (0.75 to 1.01)	1.01 (0.96 to 1.06)	0.98 (0.93 to 1[illegible]
EDSS score (each step)	**Cladribine**	0.82 (0.63 to 1.05)	1.11 (0.90 to 1.35)	1.06 (0.91 to 1[illegible]
	**S1PRMs**	0.90 (0.72 to 1.12)	1.08 (0.89 to 1.32)	1.03 (0.88 to 1[illegible]
Pre-treatment annualised relapse rate (each unit)	**Cladribine**	1.08 (1.00 to 1.18)	0.94 (0.81 to 1.09)	1.07 (0.92 to 1[illegible]
	**S1PRMs**	0.87 (0.76 to 1.01)	0.96 (0.83 to 1.11)	1.03 (0.94 to 1[illegible]

Hazard ratios (HR) with their relative 95% confidence interval (CI) were estimated by Cox proportional hazards regression analyses for each outcome of interest within each treatment group separately, adjusting for visit frequency and, where applicable, MRI scan frequency

All *p*-values ≥0.052

© 2025 Haggiag S et al. *JAMA Netw Open*.

eTABLE 4. Reasons for Treatment Suspension with Cladribine or S1PRMs

	Total	Cladribine	S1PRMs	
n	188	86	102	
Planned discontinuation	64 (34.0)	64 (74.4)	0 (0)	*
Clinical/MRI activity	48 (25.5)	3 (3.5)	45 (44.1)	*
Not reported/	31 (16.5)	10 (11.6)	21 (20.6)	
Side effects	20 (10.6)	1 (1.2)	19 (18.6)	*
Patient's decision	11 (5.9)	5 (5.8)	6 (5.9)	
Pregnancy (planned or unexpected)	8 (4.3)	1 (1.2)	7 (6.9)	
Disease progression	6 (3.2)	2 (2.3)	4 (3.9)	

Data are reported as n (%)

* $p < 0.01$ by the Chi-squared test

© 2025 Haggiag S et al. *JAMA Netw Open*.

eTABLE 5. Subsequent Therapies Following Discontinuation of Cladribine or S1PRMs

	Total	Cladribine	S1PRMs
n	188	86	102
Escalation to MABs	96	52	44
- Ocrelizumab	40	17	23
- Natalizumab	20	17	12
- Ofatumumab	29	17	3
- Alemtuzumab	6	1	5
- Rituximab	1	1	0
De-escalation	44	28	16
- Interferon Beta	17	15	2
- Dimethyl fumarate	15	7	8
- Glatiramer Acetate	10	4	6
- Teriflunomide	2	2	0
Switch to S1PRMs	6	6	0
Switch to Cladribine	5	0	5
No further DMTs reported	37	0	37

MABs: monoclonal antibodies; DMTs: disease modifying therapies

© 2025 Haggiag S et al. *JAMA Netw Open*.
