# Supplementary material for: Comparative Effectiveness of Cladribine and S1P Receptor Modulators in Treatment-Naive Relapsing-Remitting MS
Source: JAMA Netw Open. 2025 Nov 3;8(11):e2541025. doi: 10.1001/jamanetworkopen.2025.41025 (PMC12584038; doi:10.1001/jamanetworkopen.2025.41025)
Supplement: Supplement 2. — Data Sharing Statement [file jamanetwopen-e2541025-s002.pdf]

# Data Sharing Statement

Haggiag. Comparative Effectiveness of Cladribine and S1P Receptor Modulators in Treatment-Naive Relapsing-Remitting MS. *JAMA Netw Open*. Published November 03, 2025. doi:10.1001/jamanetworkopen.2025.41025

**Data**
**Data available:** No

**Additional Information**
**Explanation for why data not available:** The data that support the findings of this study were obtained from the Italian MS Register. Due to privacy regulations and governance policies of the Register, the dataset is not publicly available. Data access may be granted by the Register's scientific committee upon reasonable request and in accordance with applicable legal and ethical requirements.
